# A COVID-19 necessity or the future of medical education? An evaluation of online psychiatry tutorials for medical students

**DOI:** 10.1192/j.eurpsy.2021.1591

**Published:** 2021-08-13

**Authors:** C. Wyke, S. Butler

**Affiliations:** Department Of Undergraduate Psychiatry, South London and Maudsley NHS Foundation Trust, London, United Kingdom

**Keywords:** Education, Medical Students, virtual, tutorials

## Abstract

**Introduction:**

Following the national lockdown in the UK in March 2020 in response to the COVID-19 pandemic, we instigated regular online tutorials for fourth year medical students undertaking their psychiatry placement.

**Objectives:**

The aims of these tutorials were threefold: to ensure that students covered a range of key psychiatry topics, to enable them to have the opportunity for interactive tutorials with experienced psychiatrists and, not least, to create a sense of continuity and connection with their tutors and peers across the mental health block.

**Methods:**

Each student was allocated to a tutorial group comprising 10 – 15 medical students and a psychiatrist facilitator. These groups met weekly for 7 consecutive weeks at an agreed time for 60 – 90 minutes via an online platform and all covered the same allocated topic each week. We evaluated these groups via an online survey sent to the students following the programme.

**Results:**

The students rated the tutorials on average as 4.5/5 on whether they met the defined learning outcomes. On average the students did not consider that the virtual format made a significant difference to their learning, but this disguised a wide range of views that were expressed via a comment box.

**Conclusions:**

The evaluation of this project supports the use of virtual tutorials as a valuable learning tool but educators need to be aware that student views’ on these can be varied and so, long-term, a blend of virtual and face to face learning is most likely to meet the needs of all students.

